# Comparative Proteomics of Sera From HCC Patients With Different Origins

**DOI:** 10.5812/hepatmon.13103

**Published:** 2014-01-19

**Authors:** Jamal Sarvari, Zahra Mojtahedi, Yasuhiro Kuramitsu, Mohammad Reza Fattahi, Abbas Ghaderi, Kazuyuki Nakamura, Nasrollah Erfani

**Affiliations:** 1 Department of Bacteriology and Virology, School of Medicine, Shiraz University of Medical Sciences, Shiraz, IR Iran; 2 Institute for Cancer Research, School of Medicine, Shiraz University of Medical Sciences, Shiraz, IR Iran; 3 Department of Biochemistry and Functional Proteomics, Yamaguchi University Graduate School of Medicine, Yamaguchi, Japan; 4 Department of Internal Medicine, School of Medicine, Shiraz University of Medical sciences, Shiraz, IR Iran

**Keywords:** Hepatitis B Virus, Hepatitis C, Carcinoma, Hepatocellular, Electrophoresis, Gel, Two-Dimensional, Biological Markers

## Abstract

**Background:**

Hepatocellular carcinoma (HCC), a major fatal cancer worldwide, is induced by different etiological factors in the liver.

**Objectives:**

To gain insight into serum protein profiling of HCC with different etiologies.

**Patients and Methods:**

We subjected the sera of HBV-HCC, HCV-HCC, non-B non-C-HCC patients, and healthy volunteers to two-dimensional gel electrophoresis (2-DE) and liquid chromatography tandem mass spectrometry (LC-MS/MS).

**Results:**

We found 30 differentially expressed protein spots (≥ 1.5 fold P < 0.05) between these two analyses; of them 17 protein spots corresponding to 8 proteins were identified by MS. Transthyretin, leucine rich α-2-glycoprotein, and ficolin 3 were differentially expressed between HBV-related HCC and non-B non-C-HCC sera. Moreover, haptoglobin α-2 isoforms were decreased in HCV-HCC compared to non-B non-CHCC.

**Conclusions:**

Serum proteome analyses of HCC with different origins showed a differential protein pattern, presumably related to different hepatopathogenesis in liver induced by different agents. Further studies are required to clarify the importance of identified proteins for early diagnosis of HCC with different origins.

## 1. Background

Hepatocellular carcinoma (HCC) is the seventh most common and the third leading cause of cancer death worldwide (1). The major risk factors related to HCC are hepatitis B virus (HBV), hepatitis C virus (HCV) as viral factors and aflatoxin B1 exposure, alcohol consumption, inherited metabolic diseases, diabetes, and obesity as non-viral causes of HCC (1-3). In Iran, the annual incidence rate of HCC is very low (4, 5), and most of the cases are due to HBV infection (6). α-fetoprotein is the only widely used molecular marker for the diagnosis of HCC with different origins, but its serum level is normal in some HCC cases, and increased in some patients with chronic hepatitis (7). Most HCC cases are not candidates for surgery or transplantation due to their intrahepatic metastasis (8). For HCC survival improvement, finding more reliable diagnostic biomarkers and more efficient therapeutic targets seem to be mandatory. Studies on the liver tissues have shown differential expression of certain molecules between HBV-related HCC , HCV and non-B non-C HCC (9), implying that the discovery of a general reliable biomarker is complicated by the high degree of heterogeneity of HCC (10).

Comparative two-dimensional gel electrophoresis (2-DE) was successfully employed to uncover differentially expressed proteins in the tissue and body fluids in a variety of cancers including HCC. In this technique, thousands of proteins are separated in a single gel, and differentially expressed proteins are identified by mass spectrometry (MS). Differentially expressed proteins hold great promise as efficient cancer biomarkers. We previously identified differentially expressed proteins between the sera of HBV-HCC and HCV-HCC patients (11, 12). Data on comparison of serum protein patterns of viral and non-viral HCC are insufficient.

## 2. Objectives

The aim of our study was to identify differentially expressed proteins between viral and non-viral HCC patients by 2-DE and liquid chromatography MS (LC-MS/MS). Identification of these differentially expressed proteins may provide possible different biomarkers for early diagnosis of HCC with different origins. Moreover, such proteins may expand information related to specific mechanisms of liver pathogenesis in HCC with different origins, leading to a more efficient therapeutic strategy.

## 3. Patients and Methods

### 3.1. Subjects

Patients (N = 17) were recruited from the Department of Transplantation at Namazi Hospital in Shiraz, Iran from September 2007 to April 2009. Seven were HBV-HCC patients, 5 were HCV positive, and 5 had non-viral HCC. Their diseases were confirmed by biochemical, virological, imaging, and pathological examinations. The demographic, clinical, and laboratory characteristics of 17 HCC patients are shown in Table 1. In this study, we included 7 age- and sex-matched healthy individuals with no history of liver diseases, HBV and HCV laboratory signs, malignancies, and recent or chronic infectious diseases. The Ethics Committee of Shiraz University of Medical Sciences approved the study, and written informed consent was obtained from all participants before sampling. 

**Table 1.** Demographic, Clinical and Laboratory Characteristics of 17 HCC Patients

No.	Age	Gender	HBeAb/HBeAg	HBcAb/HCVAb	AFP a	ALT a	AST a	Cirrhosis	Child-Pugh
**1**	32	M	+/+	+/-	10.2	55	67	+	B
**2**	53	M	-/+	+/-	11.4	134	163	-	B
**3**	44	F	-/+	+/-	14.1	107	146	-	A
**4**	53	M	-/+	+/-	653	39	138	+	C
**5**	51	M	+/+	+/-	724	107	166	+	B
**6**	53	M	-/+	+/-	96.3	394	365	+	B
**7**	54	M	-/+	+/-	1.3	37	22	-	C
**8**	55	M	-/-	-/+	15	43	127	+	C
**9**	54	M	-/-	-/+	6.2	52	80	-	B
**10**	46	M	-/-	-/+	6	75	146	+	B
**11**	47	M	-/-	-/+	5	105	144	-	C
**12**	32	M	-/-	-/+	8	26	28	+	B
**13**	16	M	-/-	-/-	1.2	61	73	-	B
**14**	60	F	-/-	-/-	2.8	48	59	-	A
**15**	25	M	-/-	-/-	2.3	23	84	-	C
**16**	54	F	-/-	-/-	4.3	32	148	-	B
**17**	68	M	-/-	-/-	8.5	136	155	+	C

^a^Abbreviations: ALT, alanine aminotransferase ; AFP, α-fetoprotein ; AST, aspartate aminotransferase.

### 3.2. Serum Samples

A blood sample of 5 mL was drawn from each participant, and allowed clotting for 2 h. Then, the blood samples were spun at 3000 rpm for 10 min and the serum was separated, aliquoted and stored at -70°C until testing. High abundant proteins including albumin and Immunoglobulin G (IgG), for increasing serum protein resolution were depleted from 60 µL of the serum by Arum protein mini kit (Bio-Rad, Hercules, CA, The USA). Protein concentration of the depleted sera was determined by Bradford protein assay using albumin as the standard. 

### 3.3. 2-DE and LC-MS/MS Analysis

2-DE was performed as described previously (11). Briefly, approximately 100 µg proteins were mixed with rehydration buffer, and loaded on immobilized pH gradient (IPG) strips pH 3-10 linear (Bio-Rad) in the first dimensional isoelectric focusing. The strips were focused at 80000 Vh. The focused strips were incubated in an equilibration buffer, and then sealed on top of a 12.5% SDS. Second, dimensional electrophoresis was performed using the Protean II xi cell (Bio-Rad). Electrophoresis was run at 10 mA per gel for 30 min followed by 25 mA per gel until the tracking dye reached the bottom of the gels.

The gels were visualized by a complete protocol of a silver staining method for analytical gels. For preparative gels, the method was modified for making the standard protocol compatible with the MS analysis (13).

The silver stained gels were scanned using GS-800 calibrated densitometer (Bio-Rad). The gel images were analyzed by Progenesis software (Nonlinear, Newcastleupon Tyne, The UK) according to the instruction for finding differentially expressed proteins. Protein spots whose normalized volumes were changed more than 1.5 fold and P < 0.05 were picked up from the gels stained with the MS compatible method. 

In-gel digestion and MS were also performed as previously described (11). In brief, for LC-MS/MS analysis, the lyophilized samples were resuspended in formic acid (FA) before the LC-MS/MS analysis. An Agilent 1100 LC/MSD trap XCT was used for HPLC and MS/MS. The solutions used were water/0.1% FA and ACN/0.1% FA. A trap column (Agilent, G 1375-87320, 105 mm, 25 µm, Germany) was connected to a standard column (Zobrax 300 SB-C18, 3.5 µm, 75). Twelve microliters of the peptides was loaded on a trapping column and desalted. The elution program was 2-60 % B in 55 min, 80% B in 8 min, re-equilibration of 2% B in 10 min, for a total run of 78 min. The MS was operated in the standard scan mode for MS analysis, and in the ultra-scan mode for MS/MS analysis. The MS/MS data were analyzed with spectrum Mill (Agilent, palo Alto, CA) against the Swiss-prot database (released May, 2010). The following filters were used after database searching: peptide score 8, peptide %SPI > 70 and protein score 10. 

## 4. Results

**Figure 1.**
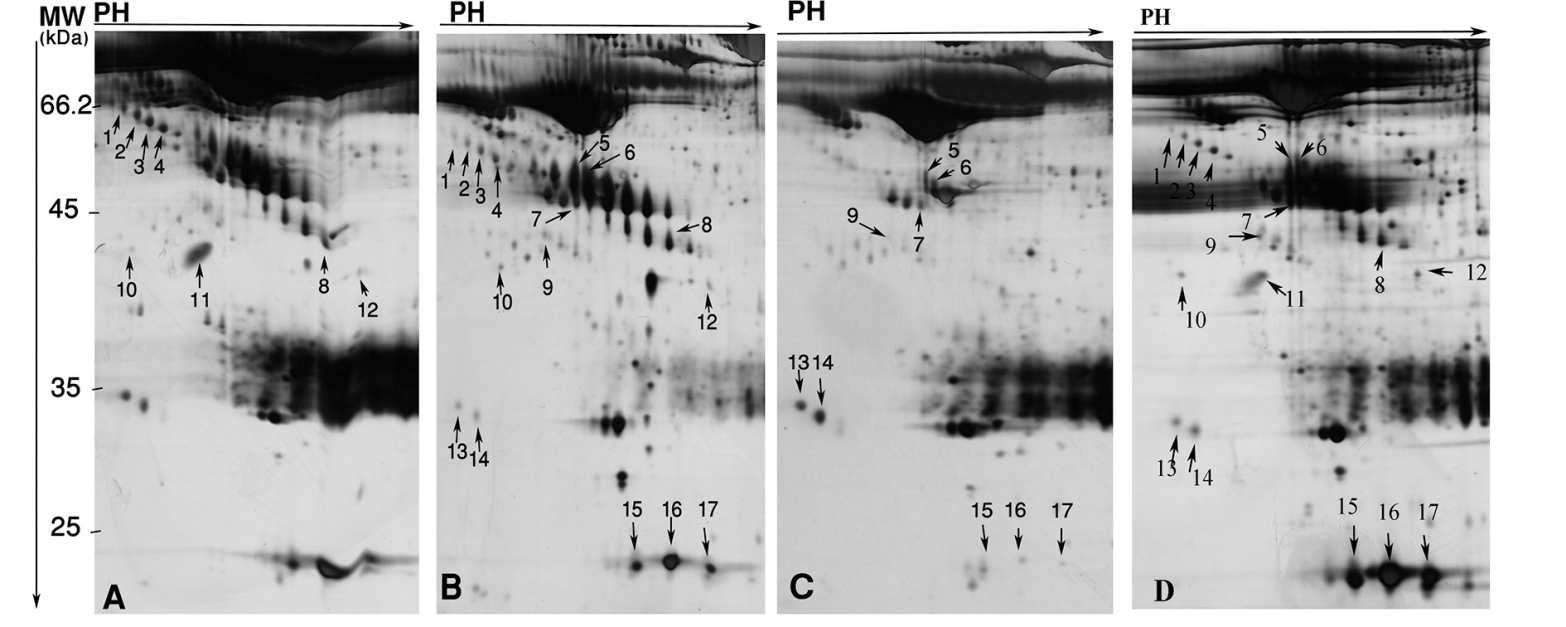
Differentially Expressed Proteins in HBV-HCC (A), HCV-HCC (B), and non-B non-CHCC (C) Patients, as Well as Healthy Volunteers (D) Sera Numbered on Corresponding 2-DE gels. Spot Numbers are for Cross Reference to the Table 2, Figures 2 and 3.

In the present study, sera of 7 HBV-HCC, 5 HCV-HCC, 5 non-B non-C-HCC, and 7 healthy volunteers were subjected to 2-DE, and differential protein spots were identified by MS. We performed two analyses between HBV-HCC and non-B non-CHCC, and HCV-HCC versus non-B non-C-HCC. Between these two analyses, 30 protein spots were differentially expressed (≥ 1.5 fold and P < 0.05) (Table 2). Of them, 17 were identified by MS. The identified spots corresponded to 8 proteins. Table 2 displays molecular weights (MW), pI, accession numbers, and proposal function of the identified proteins. The locations of these spots on the gels are shown in the Figure 1. 

**Table 2.** Seventeen Differentially Expressed Protein Spots in Sera of Viral and Non-Viral HCC Identified by LC-MS/MS, Spot Numbers are Related to the Figures 1, 2 and 3

No. Spot	Protein Name	*Mr Pre/Exp* ^*a*^ *Pre/Exp* ^*a*^	*pI Pre/Exp* ^*a*^ * Pre/Exp*	Acc ^a^ No.	Coverage	Function
1	Leucine-rich α-2-glycoprotein	38.1/48	6.45/4.3	P02750	3	Unknown
**2**	Leucine-rich α-2-glycoprotein	38.1/47	6.45/4.4	P02750	38	Unknown
**3**	Leucine-rich α-2-glycoprotein	38.1/46	6.45/4.6	P02750	24	Unknown
**4**	Leucine-rich α-2-glycoprotein	38.1/45	6.45/4.7	P02750	10	Unknown
**5**	Haptoglobin β isoform	45.2/39	6.13/5.6	P00738	14	Hemoglobin scavenger
**6**	Haptoglobin β isoform	45.2/38	6.13/5.75	P00738	30	Hemoglobin scavenger
**7**	Zinc-α-2-ycoprotein	33.8/38	5.57/5.1	P25311	51	Stimulate lipolysis
**8**	Haptoglobin cleaved-β isoform	45.2/33.5	6.13/6.2	P00738	33	Hemoglobin scavenger
**9**	α-1-antitrypsin	46.7/33	5.37/4.7	P01009	13	Protease inhibitor
**10**	Clusterin	52.4/36	5.89/5.1	P10909	5	Unknown
**11**	Transthyretin	15.8/33	5.52/5.2	P02766	54	Thyroxine hormone carrier
**12**	Ficolin 3	32.9/31	6.2/6.75	O75636	8	Innate immunity
**13**	Immunoglobulin J chain	15.5/23	4.62/4.4	P01591	44	IgA and IgM linker protein
**14**	Immunoglobulin J chain	15.5/23	4.62/4.6	P01591	31	IgA and IgM linker protein
**15**	Haptoglobin α-2 isoform	45.2/17	6.13/5.9	P00738	21	Hemoglobin scavenger
**16**	Haptoglobin α-2 isoform	45.2/17	6.13/6.2	P00738	16	Hemoglobin scavenger
**17**	Haptoglobin α-2 isoform	45.2/17	6.13/6.6	P00738	25	Hemoglobin scavenger

^a^Abbreviations: Pre, Predicated; Exp, experimental; PI, isoelectric point; Acc,accession numbers; Mr,relative molecular mass.

### 4.1. Differentially Expressed Proteins Between HBV-HCC and non-B non-C-HCC Patients

**Figure 2.**
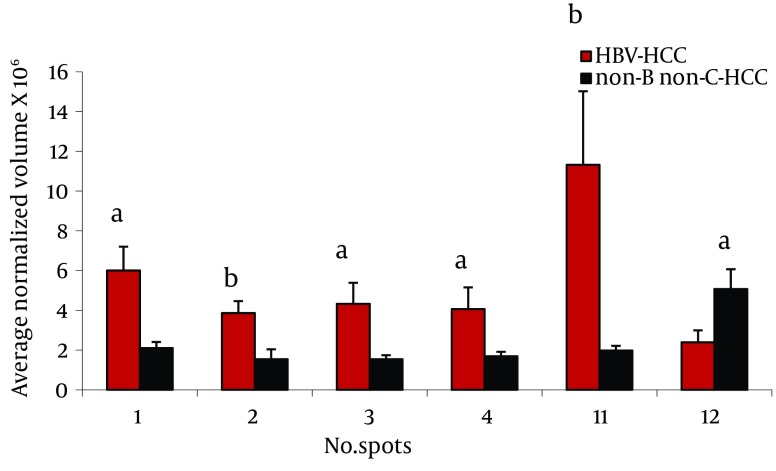
Comparison of Average Normalized Volumes of LeucineRich α-2 Glycoprotein (spots 1-4) Transthyretin (spot 11), and Ficolin 3 (spot 12) in 2-DE Gels From HBV-HCC and non-B non-CHCC Patients, as Well as Healthy Volunteers Sera, Spots Numbers Are the Same as in Table 2 and Figure 1. ^a^ P < 0.001, ^b^ P < 0.0001.

Twenty three protein spots were differentially expressed between these two groups; of them 8 including leucine-rich α-2-glycoprotein (LRG) (4 spots), transthyretin (TTR), α-1-antitrypsin (AAT), haptoglobin (HP) cleaved-β isoforms, and ficolin were identified by MS. Except ficolin 3 which its expression was decreased in HBV-HCC patients, the others were increased in these cases (Figure 2). 

### 4.2. Differentially Expressed Proteins Between HCV-HCC and non-B non-CHCC Patients

**Figure 3.**
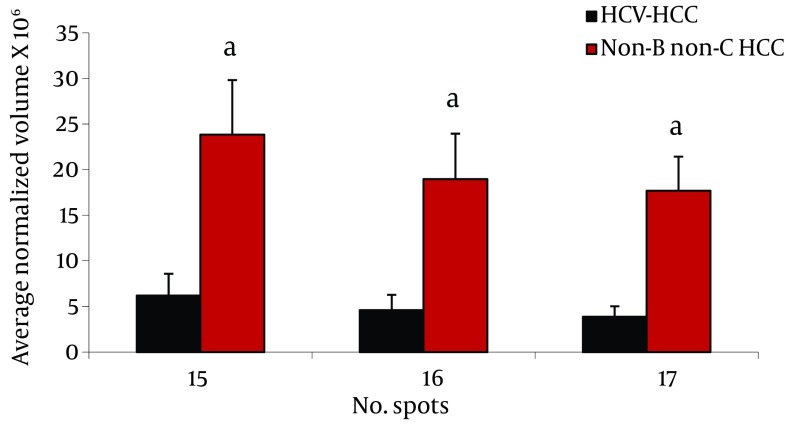
Comparison of Average Normalized Volumes of HP α-2 Isoforms in 2-DE Gels of HCV-HCC and non-B non-CHCC Patients, as Well as Healthy Volunteers Sera, Spots Numbers Are the Same as in Table 2 and Figure 1. ^a^ P < 0.01.

Analysis of serum protein expressions between HCV-HCC and non-B non-CHCC patients revealed 17 differentially expressed protein spots. Of them, 9 were identified by MS. HP α-2 isoforms (3 spots), HP β isororm (2 spots), and CLU and Zinc-α-2-glycoprotein were down- regulated in HCV-HCC compared to non-B non-C-HCC, but Immunoglobulin (Ig) J chains (2 spots) were upregulated in HCV-HCC compared to non-B non-CHCC (Figure 3). 

## 5. Discussion

Proteomics studies demonstrated a certain pattern of specificities between tissues from liver cancer associated to HBV and HCV (14), and between the sera of HCC related to HBV and HCV (11, 12). In the present study using 2DE-LC-MS/MS, the serum proteomes of HCC patients related to HBV or HCV were compared to those in non-viral HCC. 

We found differential expression (≥ 2 fold, and P < 0.05) of LRG, HP α-2 isoforms, ficolin 3, and transthyretin between these groups (Figures 2, 3). 

LRG was first isolated in the serum ( 15 ). LRG was one of the overexpressed serum proteins in HBV-HCC compared to non-B non-C HCC patients in 4 spots (Figure 2). We previously showed ( 12 ) overexpression of these protein spots in HBV-HCC compared to HCV-HCC. Increased expression of LRG has been reported in malignancies such as lung and ovarian cancers as well as bacterial and viral infection ( 16 ). In addition to liver cells as the major source of this protein, mature neutrophils and endothelial venules of the mesenteric tissues also produce it ( 16 ). Although, it is induced by IL-6, IL-1-β and TNF-α in hepatoma cells, and overexpressed in the liver of mice challenged by lipopolysaccharide rendering it as an acute phase protein, its real physiological functions has not been clarified so far ( 17 ). With the current knowledge, the reason of LRG level differences between HBV-HCC and non-B non-C-HCC is not clear. LRG is suggested as a marker for poor prognosis in HCC ( 18 ). 

We found 2 spots as TTR, one of which was multimeric form. Interestingly, this multimeric form of serum transthyretin showed an increase (6 fold) in HBV-HCC patients compared to non-B non-C-HCC patients (Figure 3). In addition, one of the monomeric forms was down-regulated (4.3 fold) in sera of patients with cirrhosis compared to CAH. TTR, or also called prealbumin, presents in the serum and cerebrospinal fluid,and is synthesized and secreted by the liver cells and the choroid plexus of the brain. Transportation of thyroxin (T4) and retinol (vitamin A) are the two significant physiological functions of TTR ( 19 ). Considering the fact that the liver is the source of serum TTR, it is reasonable to assume that the synthesis of this protein varies in the liver disease, such as cancer and hepatitis. Treatment of HepG2 cells with IL-6, IL-1 or TNF-α causes a decrease in the mRNA level of this protein ( 20 ). TTR can inhibit IL-1 production by monocytes and endothelial cells, thus showing anti-inflammatory properties ( 21 ). Our finding in 2-DE analysis suggested conformational changes of transthyretin in HCC patients related to HBV and HCV. Different expressions of TTR in the sera have been reported in SARS, dengue fever, ovarian cancer, malignant melanoma, HCC related to HBV, and in CSF in some neurological disorders such as Alzheimer, Parkinson, and schizophrenia ( 12 , 20 , 22 - 24 ). 

Ficolin 3 had more than 2.3 fold increase in non-viral HCC compared to HBV-HCC patients (Figure 2). Regarded as a serum Lectin protein, it may play an important role in the innate immunity. Moreover, ficolin specifically binds to apoptotic cells and participates in the clearance of apoptotic cells (25). Elevated levels of this protein have been reported in lung cancer, ovarian cancer and melanoma (16, 26, 27). We reported the increased expression of ficolin 3 in HBV-HCC cases compared to HBV-cirrhotic patients (11).

Isoforms of HP α-2 were our remarkable differentially expressed protein spots between HCV-HCC and non-B non-CHCC (Figure 3). HP molecule contains two types of polypeptide chains, β (heavy, 40 KDa), and α (light, α -1, 8.9 KDa or α -2, 16 KDa) (24). HP involves in Hb scavenging, inflammatory responses, immune suppression, and angiogenesis (28). Although it is mainly produced and secreted by the liver cells, it has been reported that HP is produced by cancer cells such as malignant ovarian epithelium, renal cell carcinoma, and HCC cells (29). Increased serum HP is a very frequent report in cancer, and is assumed as a marker of inflammation and tissue damage, mainly triggered by interleukins such as IL-6 and TNF-α (28). Accumulated data suggested the usefulness of serum HP, either its level or modifications, as a biomarker for cancer diagnosis irrespective of cancer type. Not only the amount but also different isoforms of HP may change in liver diseases; therefore, it has long been used for studying various liver diseases including liver cancer. In this regard, Ang et al. reported that HPs with different degrees of glycosylation are produced by HCC tissue, while other HP glycoforms are produced by normal cells (31).

 Interestingly, different studies revealed changing of HP α-2 isoforms in the sera from breast, ovary, head and neck cancers (28, 30, 31). Here, we observed that the three spots of HP α-2 isoforms were down-regulated in HCV-HCC patients compared to non-B non-C HCC. Previously, we reported the increased expression of this protein in HBV-HCC subjects compared to HBV-cirrhotic patients (11), and also in HBV-HCC compared to HCV-HCC patients (12). Although increased expression of this protein in HBV-HCC compared to HCV-HCC and non-B non-CHCC is not clear, these isoforms may have a special biological function and their occurrence may be related to an alteration of unknown intracellular process. Elevated serum levels of dissociated fragments of HP in HCC cancer, namely HP α-2 chain, has been suggested as a factor which inhibits the native HP-Hb complex and interferes with immune cellular response through its potent immunosuppressant activity (24). 

Although we have identified several differentially expressed proteins among HCC with different origins, some limitations still exist. The identified proteins should be confirmed by other techniques such as western blotting, real-time PCR, or ELISA in a larger number of patients.

In conclusion, by a proteomic approach, the sera of HBV, HCV associated HCC and non-B non-CHCC have shown different protein patterns such as increased expression of LRG in HBV-HCC compared to non-B non-CHCC, and decreased expression of HP α-2 isoforms in HCV-HCC compared to non-B non-CHCC. Further studies are required to clarify the role of the identified proteins as disease biomarkers for diagnosis, prognosis, and therapy guidelines.
